# Machine learning algorithms for identifying tuberculous pericardial effusion: insights from a retrospective study in Vietnam

**DOI:** 10.1186/s12911-026-03454-9

**Published:** 2026-03-21

**Authors:** Hai Phuong Nguyen Tran, Kha Minh Nguyen, Nam Nhat Nguyen, Vi Tuong Dang, Sang Quang Ly, Tai Nhat Nguyen, Lan Thanh Phan, Sy Van Hoang

**Affiliations:** 1 Department of Interventional Cardiology, Cho Ray Hospital, Ho Chi Minh City, 700000 Vietnam; 2 Department of Internal Medicine, Faculty of Medicine, University of Medicine and Pharmacy at Ho Chi Minh City, Ho Chi Minh City, 700000 Vietnam; 3 Department of Cardiology, Cho Ray Hospital, Ho Chi Minh City, 700000 Vietnam; 4 Present Address: Hanh Phuc International Hospital, Ho Chi Minh City, 700000 Vietnam

**Keywords:** Pericardial effusion, Tuberculosis, Machine learning, Prediction, Random tree

## Abstract

**Background:**

Tuberculous pericardial effusion (TPE) presents significant diagnostic challenges due to its nonspecific clinical presentation and similarities with other types of pericardial effusion. The available data on the use of artificial intelligence for predicting TPE is minimal and needs further expansion. This study aimed to evaluate the diagnostic performance of various machine learning algorithms (MLAs) in identifying TPE among patients with pericardial effusion.

**Materials and methods:**

A retrospective study was conducted at Cho Ray Hospital in Vietnam from 2010 to 2020. Eight MLAs—logistic regression, K-nearest neighbor, support vector machine, random forest, Lagrangian support vector machine, random tree (RT), chi-square automatic interaction detection, and C5.0—were evaluated for their diagnostic accuracy. The performance metrics included sensitivity, specificity, positive predictive value, negative predictive value, positive likelihood ratio, negative likelihood ratio, and accuracy.

**Results:**

Of the 248 patients with pericardial effusion, 52 were confirmed to have tuberculosis. Predictive factors for TPE included male sex, a lower body mass index, and fever at admission. The RT model demonstrated the highest accuracy (94%) and area under the curve (AUC) (0.971). Pericardial fluid adenosine deaminase was identified as the most significant feature for TPE diagnosis, with an optimal threshold of 27.8 U/L, a sensitivity of 80.8% and a specificity of 84.2%.

**Conclusion:**

Machine learning algorithms, particularly the random tree model, demonstrate promising potential for improving TPE diagnosis through noninvasive data analysis. However, successful implementation requires external validation and careful consideration of local healthcare capabilities.

## Background

Tuberculosis-induced pericardial effusion is still a common condition in African and Asian countries and is increasingly reported in European and Western regions [1]. Vietnam faces a substantial burden of tuberculosis. According to a World Health Organization (WHO) report in 2020, Vietnam ranks 10th among 30 countries in terms of tuberculosis burden and 11th among the 30 countries with the highest burden of multidrug-resistant tuberculosis globally [2]. In this context, tuberculosis effusion (TPE) emerges as a critical complication required special attentions. Establishing a diagnosis can be challenging and is frequently delayed or overlooked, resulting in subsequent complications such as constrictive pericarditis and increased mortality [3].

In patients with suspected tuberculosis, acid-fast bacillus staining, tuberculosis culture, or radioactive growth detection (e.g., BACTEC-460), adenosine deaminase (ADA), interferon (IFN)-gamma, pericardial lysozyme and tuberculosis polymerase chain reaction (TB-PCR) analysis should be performed [4]. However, pathogenic diagnosis using smears or cultures of pericardial fluid specimens exhibits low positivity rates or long culturing times. The bacterial culture test for tuberculosis on conventional solid media typically takes 3 to 4 weeks, while culturing on liquid media usually takes 2 weeks. Rapid tests such as direct AFB (Acid-fast bacillus) smear microscopy may be performed in a shorter timeframe but yield a lower sensitivity [5–7].

In patients with predominant lymphocytic pleural effusion, elevated ADA levels often suggest tuberculosis as the underlying cause [5]. However, it is essential to note that ADA measurement alone may not be accurate enough for diagnosing tuberculosis, especially considering that its cutoff values can vary among studies [5, 8]. Therefore, the diagnosis of TPE remains a challenge in clinical practice. Delayed or missed diagnoses can lead to severe complications, including constrictive pericarditis and increased mortality. Although some studies have examined predictive markers for tuberculosis in pericardial effusion, there remains a need for an early detection methodology that emphasizes noninvasiveness and high accuracy.

Recently, there has been increasing emphasis on utilizing artificial intelligence (AI) in medical research. Machine learning, a subset of artificial intelligence, employs algorithms and statistical models that enable computers to learn and make predictions or decisions without explicit programming. By analyzing large volumes of data, machine learning algorithms (MLAs) can identify characteristics, relationships, and anomalies that are not readily apparent [9, 10]. In diagnosing pleural effusion, MLAs can be trained to recognize subtle features in clinical data, imaging, and laboratory results that indicate the presence of the disease.

Several recent studies have demonstrated the potential of machine learning in diagnosis tuberculosis pleural effusion. Ren et al [11]. conducted a study on 1262 patients with pleural effusions to explore the use of machine learning models. They analyzed 28 different features and employed four MLAs, with the random forest (RF) model showing superior diagnostic performance sensitivity 89.1% and specificity 93.6%). Liu et al. reported that the support vector machine (SMV) was the most effective model for predicting TPE in a study involving 1435 patients [12]. Additionally, Khan et al. utilized an artificial neural network (ANN) algorithm for predicting tuberculosis in 12,636 suspected patients, achieving an overall accuracy of > 94% [13]. Despite these advances in diagnosis tuberculosis pleural effusion, there is a notable absence of studies investigating AI application specificially for TPE diagnosis, which is particularly significantly given that tuberculosis pleural effusion and TPE share many clinical features and diagnosis criteria. The successful application of machine learning in pleural effusion diagnosis suggests potential benefits for TPE detection; however, this remains largely unexplored.

In that context, our study was conducted to determine the optimal model for diagnosing tuberculous pericardial effusion (TPE) by utilizing MLAs. This research aimed to provide preliminary evidence for a novel diagnostic approach for TPE, with potential implications for improving early detection and treatment outcomes in both high-burden and resource-limited settings.

## Methods

### Subjects and study design

A retrospective study was conducted at Cho Ray Hospital in Vietnam between 2010 and 2020. The medical records of patients diagnosed with pericardial effusion were reviewed. TPE was confirmed when one of the following invasive or noninvasive diagnostic criteria was met. [1] Invasive standards included (1) positive M. tuberculosis culture from pericardial tissue or fluid, (2) a pericardial tuberculous granuloma with AFB (acid-fast bacilli) positivity, (3) a pericardial tuberculous granuloma with a positive tuberculin skin test, (4) a tuberculous pleural granuloma with AFB positivity, (5) tuberculous granulomatosis with a positive tuberculin skin test, and (6) a tuberculous granuloma in the deep carotid or peripheral lymph nodes. Noninvasive standards included (1) tuberculosis in another organ in the body, (2) mediastinal lymph nodes on a CT scan of the chest with central hypoattenuating features, a carpet pattern, and a positive tuberculin skin test, and (3) a response to TB-specific treatment. Clinical and laboratory characteristics, including demographic information, medical history, physical examination findings, laboratory test results, and imaging results. A step-by-step process is used to diagnose TPE based on invasive and non-invasive criteria. Step 1: Initial Clinical Assessment includes collecting medical history, symptoms such as chest pain and fever, and examining signs of pericardial effusion. Step 2: Laboratory Testing involves blood tests, ESR, CRP, and a tuberculin skin test (TST) to assess TB exposure. Step 3: Imaging Studies include chest X-ray, echocardiography to detect effusion, and chest CT to check mediastinal lymph nodes. Step 4: Invasive Procedures are performed if noninvasive criteria are insufficient. Pericardial fluid aspiration and biopsy (if feasible) are conducted to test for TB and AFB. Step 5: Histopathology and Microbiology Testing aim to identify TB bacteria in pericardial fluid or tissue samples. Step 6: Noninvasive Criteria Review is used if invasive criteria are unmet, including checking for TB in other organs or response to TB treatment. Step 7: Final Diagnostic Decision is made by integrating all findings. Step 8: Documentation and Treatment Initiation involve recording all information and starting TB treatment once TPE is confirmed.

### Statistical analysis

Statistical analysis was performed using SPSS version 22.0 (SPSS Inc., Chicago, IL, USA), with continuous variables presented as the mean ± standard deviation and categorical variables as frequencies. As appropriate, differences between groups were assessed using t-tests, Mann‒Whitney U tests, chi-square tests, or Fisher’s exact tests. Assumptions for normality and homogeneity of variances were checked, and nonparametric tests were used where assumptions were violated. Statistical significance was defined as *p* < 0.05. Predictor importance was evaluated based on feature importance metrics from each machine learning model, specifically utilizing measures like Gini impurity for random forest (RF) and information gain for C5.0.

### Machine learning and model validation methodology

**Fig. 1:**
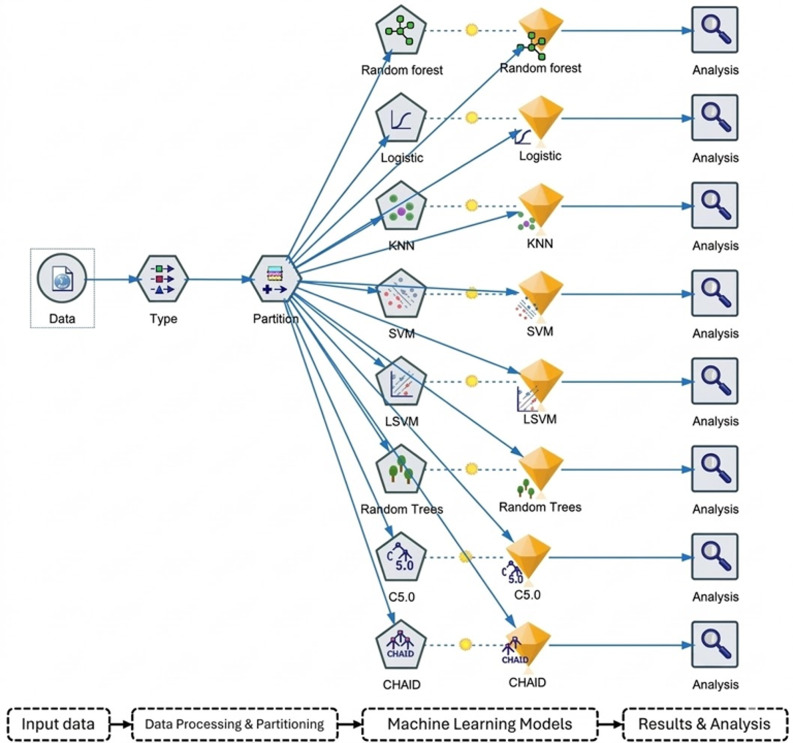
IBM SPSS model for the prediction of tuberculous pericardial effusion. Abbreviations: KNN, k-nearest neighbor; SVM, support vector machine; LSVM, Lagrangian support vector machine; CHAID, chi-square automatic interaction detection

This study analyzed multiple baseline clinical parameters, encompassing demographic characteristics (age, sex), medical history, clinical manifestations, and laboratory findings. Eight MLAs were implemented: logistic regression (LR), K-nearest neighbor (KNN), support vector machine (SVM), random forest (RF), Lagrangian support vector machine (LSVM), random tree (RT), chi-square automatic interaction detection (CHAID), and C5.0 methods (Fig. 1). Data preprocessing was requisite for LR, KNN, and SVM algorithms, incorporating mean imputation for normally distributed variables and median imputation for skewed distributions, whereas RF analysis utilized unprocessed data. The dataset underwent a 70:30 partition for training and testing sets, respectively, with implementation of 5-fold cross-validation methodology, wherein the dataset was systematically divided into quintiles, with 80% allocated to training and 20% to validation in each iteration.

To optimize model performance and mitigate overfitting, 20 iterations of model construction were executed per algorithm, incorporating early stopping criteria and regularization techniques. Model diagnostic efficacy was evaluated through comprehensive statistical analyses of confusion matrices, yielding sensitivity, specificity, predictive values (positive and negative), and likelihood ratios. Performance assessment was further quantified through receiver operating characteristic (ROC) curve analysis and area under the ROC curve (AUC) calculations. The reported performance metrics represent aggregated means across both the 5-fold cross-validation and the 20 randomized training-testing iterations, thereby ensuring robust evaluation of model reliability and generalizability in clinical application. This rigorous methodological approach enhances the validity and reproducibility of the MLAs in diagnosing TPE.

## Results

### Baseline characteristics

From January 2010 to December 2020, we recruited 248 patients with pericardial effusion who met the inclusion criteria at Cho Ray Hospital, Vietnam. Among these, 52 cases of TPE were reported. The average age was 51.0 ± 20.0 years, with males comprising 77% of the patients. Analysis of pericardial effusion etiology revealed malignancy as the predominant cause (48.4%, *n* = 120), followed by tuberculosis (21.0%, *n* = 52), idiopathic causes (10.5%), autoimmune diseases (7.3%), uremic syndrome (5.2%), and infection (4.8%).

Comparison between TPE and non-TPE groups revealed seveal significant differences in baseline characteristics Table 1. Patients with TPE were more likely to be male, have a lower body mass index, have a history of tuberculosis, and present with fever upon diagnosis. Furthermore, compared with those of patients with other causes of pericardial effusion, the serum marker levels of carcinoembryonic antigen (CEA) and white blood cell counts were lower in TPE patients. Additionally, TPE patients displayed a predominance of pericardial lymphocytes, elevated adenosine deaminase (ADA) levels, and increased lactate dehydrogenase (LDH) levels.

**Table 1:** Comparison of clinical and laboratory findings between TPE patients and non-TPE patients

Variables	TPE (*n* = 52)	Non-TPE (*n* = 196)	*p*
Male, n (%)	40 (76.9)	113 (57.7)	**0.011**
Age at diagnosis, years	47.5 (35–68)	56 (38–63)	0.432^b^
**History**,** n (%)**			
Tuberculosis	12 (23.1)	10 (5.10)	**< 0.001**
Smoking	1 (1.9)	10 (5.1)	0.467
**Symptoms at diagnosis**,** n (%)**			
Fever > 37.5 °C	21 (40.4)	27 (13.78)	**< 0.001**
Cough	24 (46.2)	74 (37.8)	0.271
Chest pain	28 (53.9)	82 (41.8)	0.121
Dyspnea	47 (90.4)	175 (89.3)	0.818
Anorexia	2 (3.9)	21 (10.7)	0.129
Fatigue	29 (55.8)	118 (60.2)	0.563
Nausea	2 (3.9)	12 (6.1)	0.527
Weigh loss	4 (7.7)	8 (4.1)	0.281
BMI (kg/m^2^)	20.6 ± 3.1	21.6 ± 3.2	**0.035**
**Laboratory tests**			
***Blood***			
White blood cells (G/L)	8.0 (6.3–9.9)	10,6 (7.3–14.4)	**< 0.001** ^**b**^
Hemoglobin (g/L)	117.0 (109.0–130.0)	112.5 (97.2–128.0)	0.099^b^
Platelets (G/L)	277.0 (222.0–380.5)	267 (202.0–349.0)	0.406^b^
C-reactive protein (mg/L)	6.5 (6.5–37.6)	6.5 (6.5–32.7)	0.892^b^
Glucose (mg/dL)	103.5 (91.0–122.5)	106.0 (89.0–131.0)	0.668^b^
LDH (U/L)	492.7 (293.5–710.5)	418.0 (261.0–711.0)	0.260^b^
Protein (g/dL)	6.1 (5.6–6.8)	5.9 (5.4–6.5)	0.154^b^
Albumin (g/dL)	2.4 (2.2–2.8)	2.4 (2.2–3.1)	0.730^b^
Blood urea nitrogen (mg/dL)	13.0 (10.5–18.5)	17.0 (12.0–31.5)	**0.002** ^**b**^
Creatinine (mg/dL)	0.97 (0.8–1.1)	1.04 (0.8–1.3)	0.063^b^
eGFR (mL/min/1,73m^2^)	88.3 ± 28.0	72.9 ± 32.8	**0.002**
Sodium (mmol/L)	135.0 (130.0–138.0)	135.0 (130.5–137.0)	0.978^b^
Adjusted CEA, (ng/mL)	2.4 (1.2–2.9)	2.9 (2.9–4.9)	**< 0.001** ^**b**^
***Pericardial fluid***			
Bloody effusion, n (%)	24.0 (46.2)	127.0 (64.8)	**0.014**
White blood cells, n (%)			0.517
Low (< 100/mm^3^)	6.0 (11.5)	35.0 (17.8)	
Medium (100–1000/mm^3^)	13.0 (25.0)	40.0 (20.4)	
High (1000–10 000/mm^3^)	31.0 (59.6)	107.0 (54.6)	
Very high (> 10 000/mm^3^)	2.0 (3.9)	14.0 (7.1)	
Neutrophils (%)	18.0 (10.0–50.5)	24.0 (14.0–52.5)	0.449
Lymphocytes (%)	55.5 (30.0–84.0)	30.0 (16.0–59.0)	**< 0.001**
Red blood cells, n (%)			**0.005**
Low (< 100/mm^3^)	10.0 (19.2)	44.0 (22.4)	
Medium (100–1000/mm^3^)	10.0 (19.2)	9.0 (4.6)	
High (1000–10 000/mm^3^)	21.0 (40.4)	82.0 (42.3)	
Very high (> 10 000/mm^3^)	11.0 (21.2)	59.0 (30.4)	
Mesothelial cells (%)	5.5 (2.0–15.0)	15.0 (3.0–32.0)	**0.009**
Protein (g/dL)	5.1 (4.5–5.6)	4.9 (4.1–5.5)	0.067
Albumin (g/dL)	2.4 (2.2–2.8)	2.4 (2.2–3.1)	0.730
Exudative effusion, n (%)	52.0 (100.0)	191.0 (97.5)	0.107^a^
Glucose (mg/dL)	60.5 (16.5–91.0)	76.0 (37.5–107.0)	0.054^b^
LDH (U/L)	2019.0 (941.5–3787.5)	1147.5 (475.0–2315.4)	**0.002** ^**b**^
ADA (U/L)	45.1 (32.1–70.1)	13.8 (6.8–24.5)	**< 0.001** ^**b**^
Malignant cells, n (%)	0 (0)	24.0 (16.2)	**0.003** ^**c**^

The data are presented as the mean (SD), median (interquartile range), or n (%)

Abbreviations: ADA, adenosine deaminase; BMI, body mass index; CEA, carcinoembryonic antigen; eGFR, estimated glomerular filtration rate; LDH, lactate dehydrogenase

### Machine learning algorithms

**Fig. 2:**
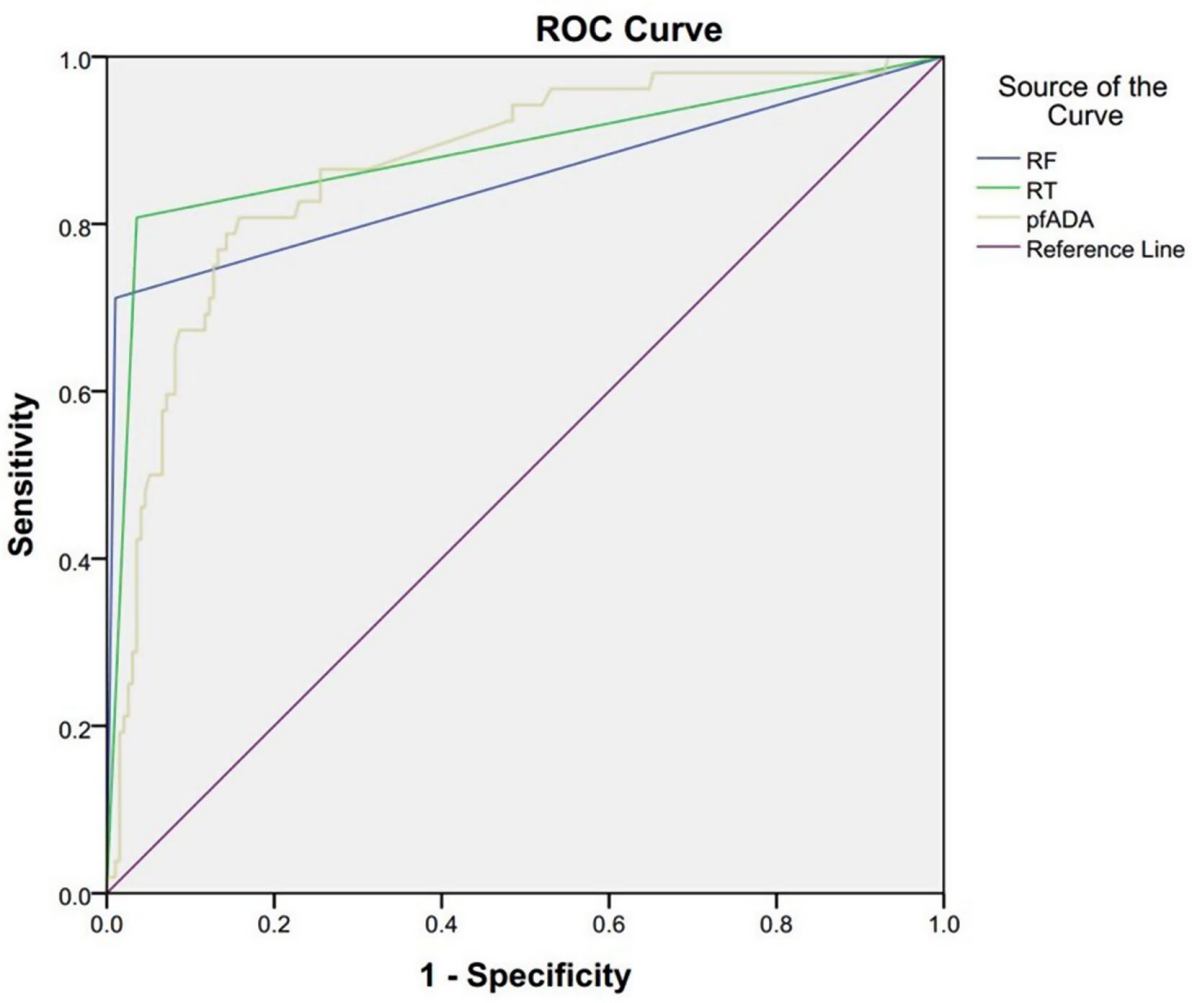
ROC curves comparing diagnostic performance of RT, RF, and pfADA for tuberculous pericardial effusion

To assess the diagnostic performance of the eight MLAs, we compared them with the conventional pericardial fluid ADA (pfADA) test (Table 2). The pfADA test, at an optimal cutoff of 27.8 U/L determined by maximizing Youden’s index (Sensitivity + Specificity − 1), achieved an AUC of 0.869 and an accuracy of 83.5%. Notably, the random tree (RT) model exhibited the highest AUC (0.971) and accuracy (94.0%), followed by the random forest (RF) model (AUC 0.968, accuracy 93.5%) (Fig. 2).

**Table 2:**
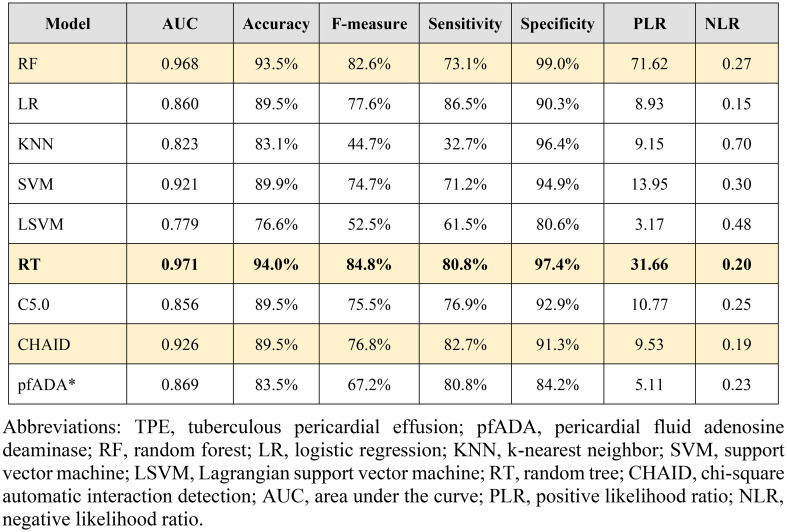
Performances of the algorithmic models and pfADA for diagnosing TPE

**Fig.3:**
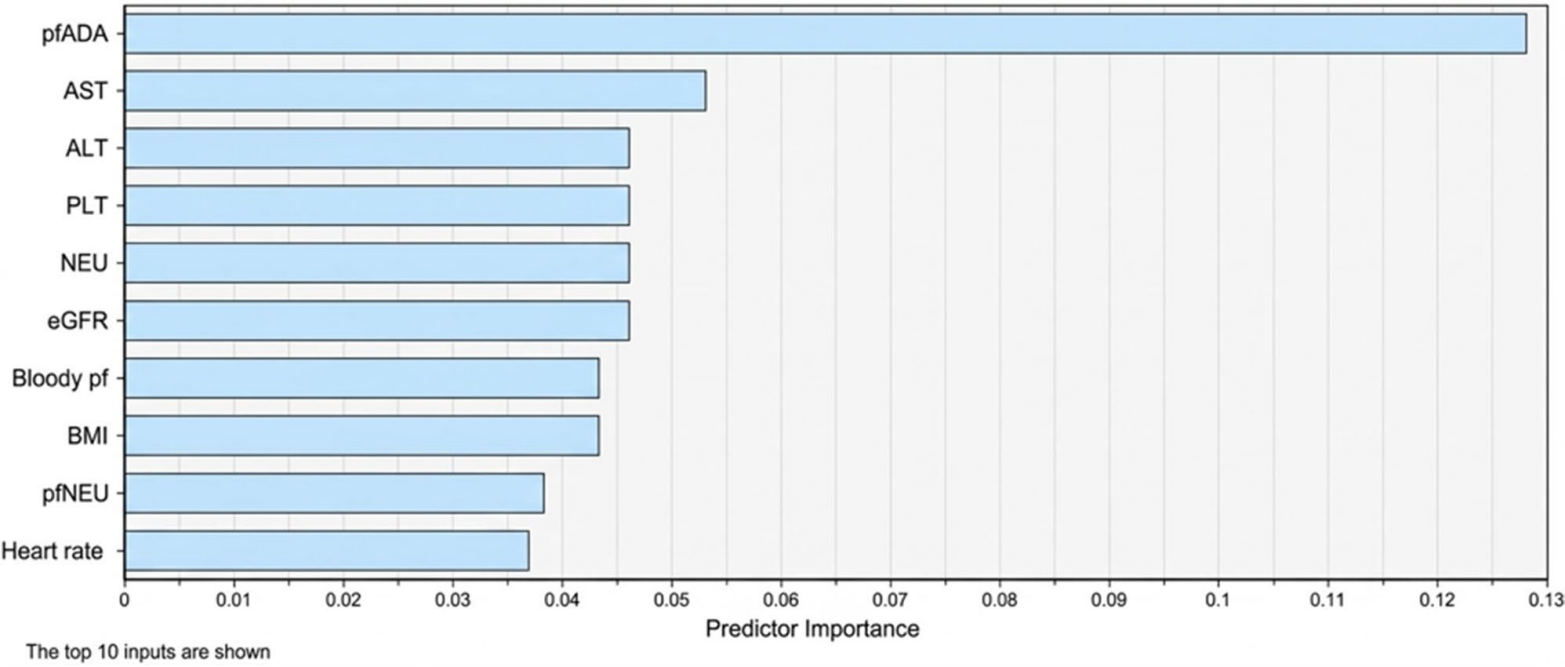
The impacts of the top 10 features on the accuracy of the RF model

We analyzed the impact of the characteristics on the model’s accuracy using the average reduction values of the Gini index. Among the top ten features, pfADA was the most crucial (Fig. 3).

## Discussion

Our study presents a systematic approach to distinguishing TPE from alternative causes of pericardial effusion. Through the application of a random tree classifier that integrates both clinical and laboratory data, we achieved enhanced diagnostic performance compared to relying on individual parameters. This methodology has significant implication for improving diagnostic accuracy and facilitating prompt treatment initiation. It is frequently challenging to isolate the causative organism to establish a definitive diagnosis of TPE. Consequently, numerous investigations have been undertaken to identify alternative diagnostic modalities for tuberculosis that offer efficient results. The measurement of ADA levels in various fluids, including pleural and pericardial effusions, has emerged as a promising diagnostic tool for TPE due to its affordability, widespread availability, and noninvasive nature [14]. Nonetheless, the sensitivity and specificity of ADA testing exhibit variability and are contingent upon chosen cutoff values [15].

Although there is evidence supporting the use of MLAs for diagnosing tuberculosis-induced pleural effusion, there is a scarcity of data on their effectiveness in diagnosing pericardial effusion. Our study illustrated the feasibility and practicality of employing MLAs to differentiate TPE from other causes of pericardial effusion. In machine learning, successful algorithm selection is crucial for optimal performance. There are four primary classification tasks exist: binary, multiclass, multilabel, and imbalanced classifications. In binary classification, the objective is to categorize input data into two mutually exclusive groups. LR and SVM algorithms are inherently tailored for binary classifications. [16] As a supervised learning algorithm, SVM demonstrates proficiency in detecting nuanced patterns within intricate datasets. Predominantly employed for classification purposes, SVM is predominantly employed for classification purposes and identifies the optimal decision boundary within high-dimensional spaces [17, 18]. In our study, SVM demonstrated the lowest accuracy and specificity among the eight alternative algorithms, while its sensitivity, though not the lowest, remained generally low. This finding parallels Ren et al.‘s study [11], wherein SVM exhibited an accuracy of 80.4%, the lowest among the four algorithms, with a specificity of 85.9% and sensitivity of 83.2%. Recently, Liu et al.‘s [12] study underscored SVM as the optimal model for discriminating tuberculous pleural effusion from nontuberculous pleural effusion, achieving a balanced accuracy of 87.7% and an AUC of 0.914. This investigation included 1435 patients who presented with pleural effusion, and the authors meticulously compared the diagnostic efficacy of nine distinct MLAs to determine the most efficient diagnostic model for TPE. Moreover, pleural fluid ADA, pleural fluid CEA, and serum CYFRA 21.1 were identified as the top three crucial features for tuberculosis diagnosis. The performance of the SVM model depends on the dataset represented, including the original distribution and its size [19, 20]. Therefore, the variation in dataset composition between our study and that of Ren et al., compared to Liu et al., may have contributed to the observed variations in the results. Garcia-Zamalloa et al. [21] conducted a prospective study involving 230 consecutive patients with pleural effusion. Their findings revealed that SVM exhibited the highest predictive capability for tuberculous pleural effusion, achieving an AUC of 0.98, an accuracy of 97%, a sensitivity of 91%, and a specificity of 98%. Nevertheless, it is imperative to note that this investigation was conducted within a low-tuberculosis-incidence setting, which contrasts with the epidemiological landscape of tuberculosis prevalent in countries such as Vietnam.

The second algorithm that can be employed for binary classification is LR. As a baseline supervised MLA, LR operates as a discriminative classifier. Discriminative models can distinguish between classes with a limited need to understand their underlying characteristics extensively. Within medical diagnostics, logistic regression has been widely applied. Shu et al. [22] utilized LR modeling to diagnose tuberculous pleural effusion, leveraging measurements of pleural fluid ADA, interferon-𝛾 (IFN-𝛾), decoy receptor (DcR) 3, and soluble tumor necrosis factor receptor 1 (TNF-sR1). Their model achieved a sensitivity of 82.9% and a specificity of 86.7%. However, since TFN-𝛾, DcR3, and TNF-sR1 are not routinely assessed in clinical settings, the applicability of the Shu et al. model is limited. Ren’s research analyzed 36 features, including clinical, routine blood laboratory, and fluid laboratory measurements [11]. The LR model yielded an accuracy of 82.9% and an AUC of 0.876, with a sensitivity of 80.5% and a specificity of 84.8%. Ren’s findings indicate that the performance of the LR in diagnosing tuberculosis falls short of traditional pleural fluid ADA testing. Our study, which achieved an AUC of 0.860 and an accuracy of 89.5%, slightly outperformed the prediction of pfADA, which had an AUC of 0.869 and an accuracy of 83.5%, in contrast to Ren’s findings. However, it is important to highlight that the logistic regression model consistently demonstrated elevated accuracy, sensitivity, and specificity.

KNN can also serve as a valuable tool for binary classification tasks [16]. Chen et al. [23] utilized the KNN approach to differentiate between normal and abnormal respiratory sounds. In an ideal acoustic setting devoid of human interference, the method achieved a perfect discrimination rate of 100%. Although replication of the study in a real-world acoustic environment is pending, these findings underscore the significant promise held by the KNN method. However, in tuberculosis diagnosis, KNN exhibits lower sensitivity, as observed in our study and studies conducted by Ren and Liu [11, 12]. Furthermore, the AUC, accuracy, and specificity of the KNN algorithm were inferior to those of other MLAs in predicting tuberculosis. This disparity in performance can be attributed to the classification characteristics of KNN. Unlike LR and SVM, which are commonly called eager learners for their immediate model construction from training datasets to enhance generalization during learning, KNN is categorized as lazy. Unlike eager learners, KNNs need to construct a model from the training data promptly. Instead, it memorizes the training dataset, and when a prediction is needed, it searches for the nearest neighbor within the entire training dataset [24].

In addition, RF represents a learning algorithm adept at handling both regression and classification tasks. By combining multiple decision trees, an RF operates by sampling subsets of observations with varying features from a given dataset [25, 26]. Similarly, a RT employs ensemble classification, making predictions by averaging over several independent base models. Previous studies have demonstrated that RF performs better for classifying various diseases. Chen et al. utilized SVM, naive Bayes, KNN, and RF as MLAs to develop decision-support systems for diagnosing liver fibrosis [27]. They found that RF yielded the highest accuracy among these MLAs. Similarly, Chicco et al. compared various MLA models, including probabilistic neural networks, perceptron-based neural networks, RF, one rule (OneR), and decision tree classifiers, for predicting pleural mesothelioma [28]. Their results demonstrated that RF outperformed all other MLA models in terms of predictive accuracy. Xiao et al. utilized the RF method to construct a diagnostic model targeting prostate cancer, yielding an accuracy of 83.1%, a sensitivity of 65.6%, and a specificity of 93.8% [29]. In a study conducted by Casanova et al., logistic regression and RF diagnostic capabilities were compared for diagnosing diabetic retinopathy. Their findings highlighted RF’s superior classification accuracy in this context [30]. Thus, RF has emerged as a notably advantageous tool for disease diagnosis applications. In our study, slight disparities were observed in the diagnostic performance of RF and RT in TPE. In our study, slight disparities were observed in the diagnostic performance of RF and RT in TPE. The AUC, accuracy, sensitivity, and specificity of RF and RT were 0.968, 93.5%, 73.1%, 99.0%, and 0.971, 94%, 80.8%, and 97.4%, respectively. Moreover, RF and RT emerged as superior methodologies to LR, KNN, SVM, LSVM, CHAID, C5.0, and pfADA. Both RF and RT demonstrated balanced performance across the AUC, accuracy, and specificity metrics. Similarly, in a study by Ren et al.,^11^ the utilization of four MLAs suggested RF as the optimal model for diagnosing TPE, with an accuracy of 91.6%, an AUC of 97.1%, a sensitivity of 89.1%, and a specificity of 93.6%. The discrepancies in sensitivity between the two studies may stem from variations in the total sample size and the exclusion of pleural effusion caused by transudation by Ren et al., potentially introducing statistical bias. Additionally, disparities in the type and number of input features (28 versus 40) further distinguished the methodologies between the two investigations. Previous reports on the use of machine learning algorithms (MLAs) for disease prediction suggest that incorporating various AI models could enhance accuracy and assist in selecting the most suitable model [31].

Given the superior accuracy and AUC of RT, we analyzed the impact of various characteristics on model accuracy using average reduction values of the Gini index. Among the ten identified factors with varying degress of influence, pfADA emerged as the most significant predictor. This finding aligns with research by Ren et al.,^11^ emphasizing the crucial role of ADA testing in tuberculosis diagnosis. The optimal threshold of 27.8 U/L in our study was determined using ROC curve analysis to maximize both sensitivity and specificity. This threshold is notably lower than the conventional cutoff of 40 U/L reported in previous studies, which showed sensitivity of 83–93% and specificity of 78–97% in diagnosing tuberculosis pericarditis [32]. The lower threshold in our study might reflect regional variations in disease presentation or differences in testing methodology, highlighting the importance of establishing location-specific reference values.

The integration of clinical factors and additional blood and fluid tests, which are routinely available in many areas, into machine learning models can enhance both the efficiency and accuracy of diagnosing TPE. This approach offers a less invasive and more accessible method for tuberculosis detection, potentially reducing the need for costly and invasive tests such as IFN-𝛾, PCR, culture, or biopsy. However, validation across diverse populations and healthcare settings remains essential before widespread implementation of this approach.

### Strengths and innovations

Our study has notable strengths that underscore its robustness. First, we integrated diverse features, including clinical indicators, blood parameters, and pericardial fluid data, into our machine learning models. This comprehensive approach enhanced the model’s ability to discern patterns and accurately diagnose TPE. Second, our systematic comparison of multiple classifier algorithms provides valuable insights into their relative performance in this specific clinical context. Additionally, by incorporating readily available clinical and laboratory parameters, we developed an approach that could be particularly valuable in various healthcare settings.

The implementation of MLAs in clinical offers potential advantages over traditional diagnostic methods. While procedures such as pericardiocentesis and biopsy remain valuable diagnostic tools, they are invasive and carry inherent risks. MLAs provide a complementary approach that can analyze multiple clinical and laboratory parameters simultaneously, potentially reducing the need for invasive procedures in some cases. This could be particularly beneficial in resource-limited settings where advanced diagnostic techniques may be less accessible.

### Limitations

Our study has several notable limitations. First, its single-center design at Cho Ray Hospital may limit the generalizability of our findings to diverse tuberculosis epidemiological settings and healthcare systems. Although we employed cross-validation and repeated train/test splits to enhance model robustness, the validation process remained confined to the same dataset population, potentially impacting the external applicability of our machine learning models. Second, the inherent challenges of working with smaller datasets introduce risks of overfitting and increased complexity in model interpretation. While we took steps to mitigate these issues, they remain important considerations in the context of machine learning-based diagnostics. Third, the retrospective nature of the study introduces potential selection biases and limits our ability to assess temporal changes in clinical parameters. Lastly, the absence of external validation restricted our ability to evaluate the real-world performance and generalizability of our models. Future studies incorporating multi-center validation and prospective evaluation will be essential to strengthen the clinical applicability of our findings.

## Conclusion

This study revealed that the potential of machine learning algorithms, particularly the random tree model, in enhancing tuberculous pericardial effusion diagnosis through noninvasive data analysis. These models could assist clinicians, especially in resource-limited settings, by providing a data-driven approach to diagnosis. However, successful implementation requires external validation across diverse populations and careful integration into clinical workflows.

## Data Availability

The datasets analysed during the current study are not publicly available. Inquiries regarding datasets can be directed via email to: hoangvansy@ump.edu.vn.

## Abbreviations

TPE: Tuberculous pericardial effusion
PE: Pericardial effusion
AFB: Acid-fast bacillus
AUC: Area under the curve
ADA: Adenosine deaminase
pfADA: pericardial fluid Adenosine deaminase
TB: Tuberculosis
MLAs: Machine learning algorithms
RT: Random tree
LR: Logistic regression
KNN: K-nearest neighbors
SMV: Support vector machine
RF: Random forest
ANN: Artificial neural network
LSVM: Lagrangian support vector machine
CHAID: Chi-square automatic interaction detection
ROC: Receiver operating characteristic
